# Cefepime Neurotoxicity in a Patient With Acute Tubular Necrosis

**DOI:** 10.7759/cureus.9911

**Published:** 2020-08-21

**Authors:** Niranjan Ojha, Sana Riaz, Ambika Eranki

**Affiliations:** 1 Internal Medicine, State University of New York Upstate Medical University, Syracuse, USA; 2 Infectious Disease, State University of New York Upstate Medical University, Syracuse, USA

**Keywords:** cefepime, neurotoxicity, elderly, renal impairment, nephrotoxic drugs, acute tubular necrosis

## Abstract

Neurotoxicity is a rare side effect of Cefepime use. Cefepime can cross the blood-brain barrier and can be neurotoxic by competitive albeit weak antagonism of the gamma-aminobutyric acid complex. It is cleared by the kidneys which puts individuals with renal impairment at risk of side effects. We describe a case of Cefepime neurotoxicity in the context of nephrotoxicity secondary to the use of other drugs.

## Introduction

Cefepime, a widely used antibiotic, is a fourth-generation cephalosporin whose microbicide action ranges from gram-positive to gram-negative organisms including Pseudomonas. Neurotoxicity is a rare side effect of cefepime use. The clinical trial for the safety of cefepime has shown neurotoxicity in 0.15% cases [1]. It can cross the blood-brain barrier and can be neurotoxic by competitive albeit weak antagonism of the gamma-aminobutyric acid complex [2,3]. Renal dysfunction is one of the risk factors in patients with signs of cefepime neurotoxicity [4].

Acute kidney injury is a major complication associated with vancomycin treatment. It causes dose-dependent acute tubular necrosis (ATN) secondary to oxidative stress in proximal tubule cells, autophagy, and obstructive cast formation. It can also cause acute interstitial nephritis [5].

## Case presentation

A 67-year-old male with a past medical history significant for type II diabetes mellitus, hypertension, and peripheral artery disease was referred to hospital by his primary care provider for non-healing Wagner grade III right foot ulcer and MRI (magnetic resonance imaging) findings of osteomyelitis involving the distal two-third of the second metatarsal and proximal second phalanx, distal head of the third metatarsal and base of the third proximal phalanx. He had a long-standing draining wound but denied pain, fever, and chills. He was started empirically on vancomycin and piperacillin/tazobactam on admission. He underwent ray amputation of the second and third toe on day 1 of admission. He developed acute kidney injury with increasing creatinine levels on day 3 of admission. The antibiotics were changed to vancomycin, cefepime, and oral metronidazole, awaiting culture.

**Figure 1:**
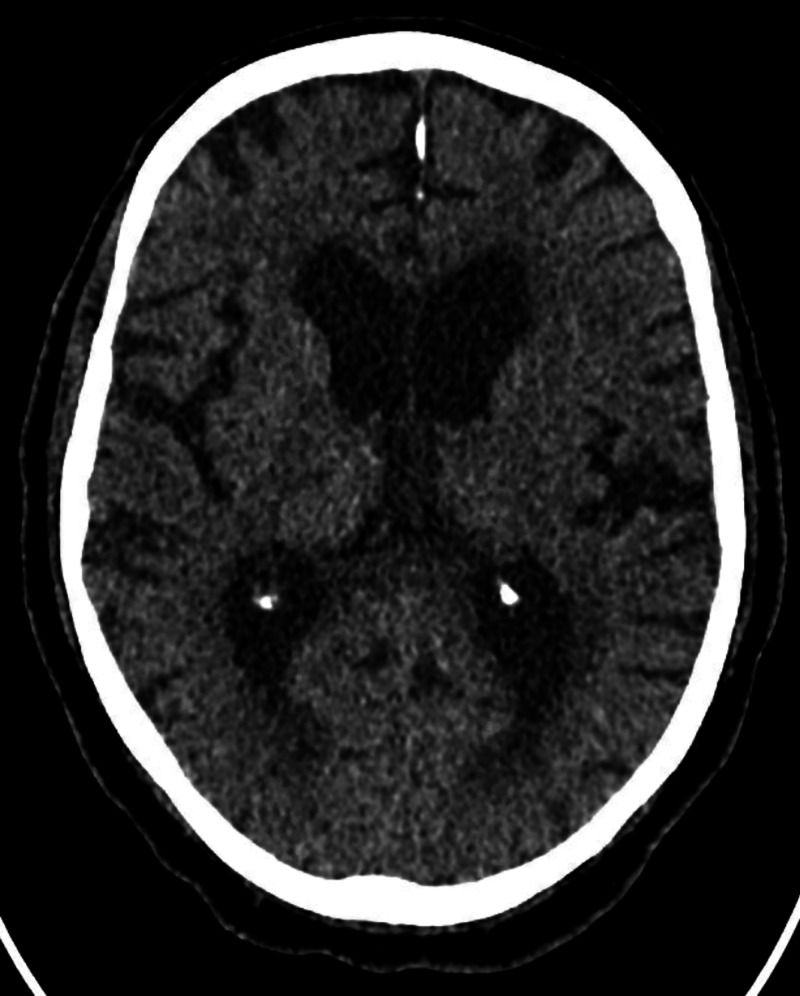
CT head without contrast showing no acute intracranial bleed or ischemia

**Figure 2:**
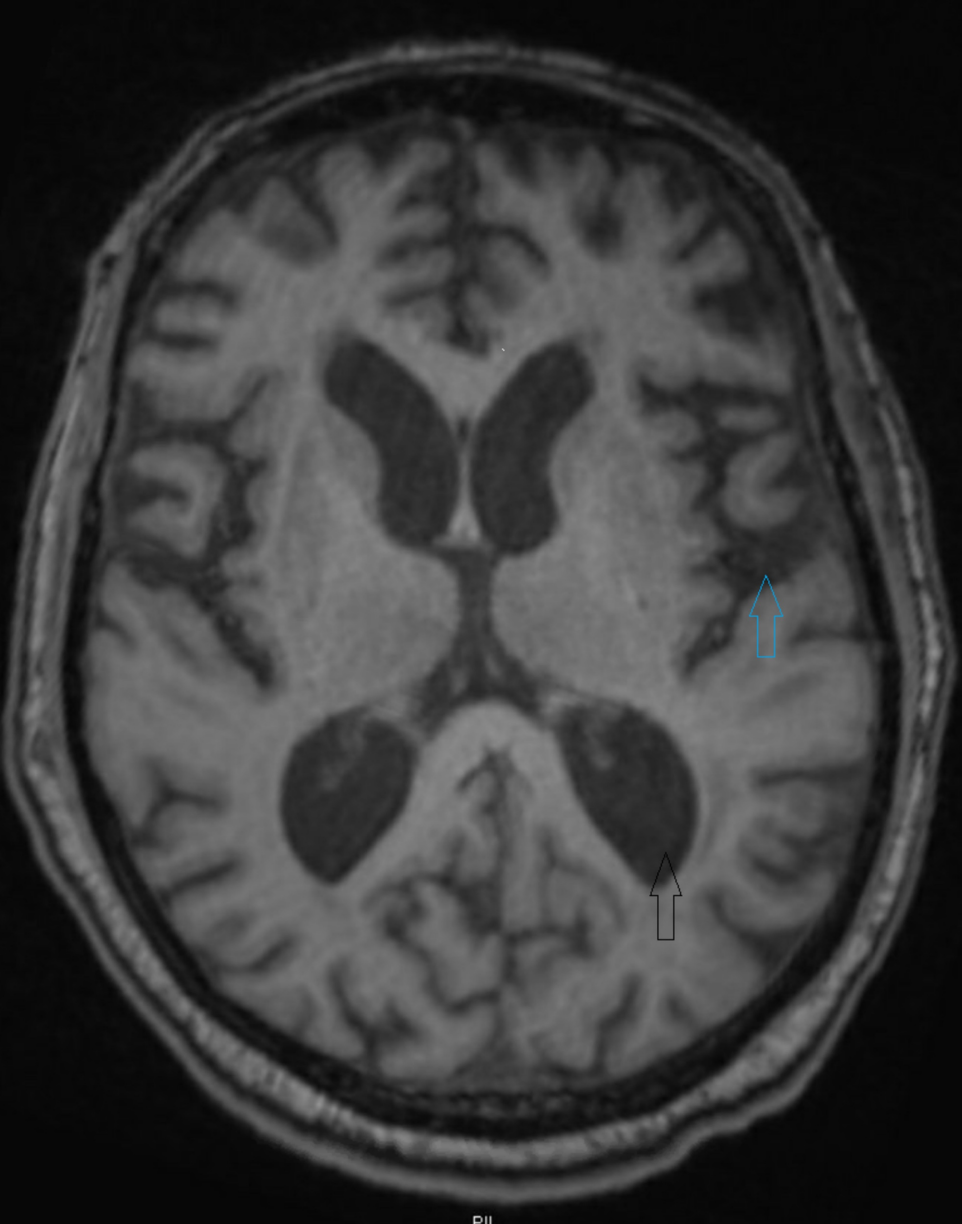
MRI brain without contrast showing age-related mild volume loss(upper blue arrow) and prominent ventricles(lower black arrow).

The patient had altered mental status on day 6 of admission and day 3 of cefepime treatment. He was initially confused and agitated. On examination, he was confused, oriented to self. He elicited asterixis, myoclonus, and hyperreflexia, and then became lethargic and stuporous. We considered worsening infection/sepsis, stroke, and hyperammonemia/uremia as differential diagnoses. His vitals showed blood pressure of 125/57 mm of mercury, pulse rate of 86 beats per minute, respiratory rate of 18 breaths per minute, and temperature of 98.1-degree Fahrenheit. His complete blood count was significant for WBC of 13.2.and complete metabolic profile was significant for Blood Urea Nitrogen of 27 mg/dl, serum creatinine was 7.59 mg/dl. His serum ammonia level was 30 micromol/liter. His urine showed muddy brown casts suggestive of acute tubular necrosis. The computed tomography (CT) head without contrast (figure 1) didn’t show any intra-cranial bleed or ischemia. The intra-operative culture grew Methicillin-Resistant Staphylococcus aureus, Enterococcus faecalis, and Bacteroides. The blood culture remained sterile for more than 5 days. Due to altered mental status, we stopped Cefepime. We switched vancomycin to daptomycin and started renally adjusted piperacillin/tazobactam due to acute kidney injury. The surgical pathology showed viable margins and we stopped all forms of the antibiotics. The patient's mentation didn’t improve for the next 3 days of stopping cefepime. Magnetic resonance imaging (MRI) of his brain (figure 2) showed age-related volume loss and prominent ventricles. From day 4 of cessation of cefepime use, he showed some improvement in his mentation which coincided with improving kidney function. He started responding to some of the questions. His mentation progressively improved over the next few days. He no longer had asterixis, myoclonus, and hyperreflexia. He was able to communicate his wishes and can make his own decisions at the time of discharge.

## Discussion

The treatment of osteomyelitis warrants the use of broad-spectrum antibiotics before a definitive culture result [6]. We started our patient on vancomycin and piperacillin/tazobactam on admission. Vancomycin is a nephrotoxic drug [5]. The nephrotoxicity of vancomycin and piperacillin/tazobactam combined is higher than that of vancomycin alone [7]. This is a standard treatment choice for broad-spectrum coverage in osteomyelitis [6]. Our patient developed acute kidney injury following the use of vancomycin and piperacillin/tazobactam as evidenced by increasing serum creatinine level and decreased glomerular filtration rate (GFR). A meta-analysis reported higher kidney injury rates with the use of vancomycin-piperacillin/tazobactam compared to vancomycin-meropenem and vancomycin-cefepime combinations [7]. Subsequently, we changed the antibiotics regimen to vancomycin, cefepime, and metronidazole.

Cefepime is a fourth-generation cephalosporin with a spectrum of coverage including gram-positive organisms, gram-negative organisms, and Pseudomonas [8]. It is cleared by the kidneys and has an elimination half-life of 2 hours which is dose-independent. It is eliminated mainly by glomerular filtration. Glomerular filtration excretes more than 80% of the administered dose (intravenous or intramuscular) unchanged in the urine in the normal functioning kidney which significantly decreases in individuals with a creatinine clearance of <30 ml/kg/m^2^ [9,10,11]. A linear relationship exists between the renal clearance of Cefepime and the creatinine clearance of an individual. The creatinine clearance decreases with age and in conditions of renal impairment making the elimination half-life significantly longer in the elderly and individuals with renal insufficiency [11,12].

Cefepime may be neurotoxic in a vulnerable population. The risk factors for cefepime neurotoxicity include the elderly population, those with renal impairment, and individuals with prior neurological disease [13]. The age of our patient put him in the risk of neurotoxicity. His serum creatinine was 2.76 mg/dl and the GFR calculated by the CKD-EPI (Chronic kidney disease epidemiology collaboration) method was 22 ml/min/1.73 m^2^, which put our patient at increased risk of neurotoxicity. The time of onset of symptoms of neurotoxicity following the use of cefepime has been different in different individuals, the median being 4 days [4].

Cefepime is a beta-lactam antibiotic which can cross the blood-brain barrier and causes neurotoxic effects by inhibiting GABA mediated neurotransmission. Another potential mechanism of neurotoxicity includes the enhancement of glutaminergic activity [13]. The common signs and symptoms of cefepime neurotoxicity include altered mental status, reduced consciousness, confusion, myoclonus, aphasia, agitation, and seizures [4]. The time of onset of neurological symptoms in our patient was 3 days and progressed over the next 2 days. Our patient exhibited similar signs and symptoms reported in the literature elsewhere but did not have a seizure.

The discontinuation of the cefepime is usually the treatment in this condition. The clinical resolution of symptoms usually occurs within 2 days of discontinuation of the drug [4]. Cefepime is dialyzable and hemodialysis decreases the elimination half-life from 13.5 hours in pre-dialysis patients to 2.3 hours after dialysis [11]. We were deciding on starting hemodialysis when the patient started showing improvement in his mentation. It took 3 days from discontinuation of cefepime for our patient to show signs and symptoms of improvement and complete recovery took 3 more days. His improvement coincided with improving renal function, creatinine clearance, and GFR. Our case highlights the importance of clinical vigilance in elderly patients while initiating antibiotics, as they are prone to more adverse effects.

## Conclusions

Cefepime is a the widely used antibiotic that rarely causes neurotoxicity. It is renally cleared, so we must be careful using it in individuals with renal insufficiency. Our case highlights the importance of clinical vigilance in elderly patients while initiating antibiotics, as they are prone to more adverse effects.
